# The additional value of radiology reports in the follow-up of extremity fractures: a retrospective study in an academic hospital

**DOI:** 10.1016/j.jcot.2025.103043

**Published:** 2025-05-02

**Authors:** Mare M.H. Walraven, Pieta Krijnen, David A. Vuijk, Monique Reijnierse, Inger B. Schipper, Marco F. Termaat

**Affiliations:** a Leiden University Medical Center – Department of Trauma Surgery, Albinusdreef 2, 2333, ZA, Leiden, the Netherlands; b Acute Care Network West Netherlands, Rijnsburgerweg 10, 2333, AA, Leiden, the Netherlands; c Leiden University Medical Center – Department of Radiology, Albinusdreef 2, 2333, ZA, Leiden, the Netherlands

**Keywords:** Fracture healing, Extremity fractures, Radiology report, Radiograph, Interrater reliability

## Abstract

**Background:**

Radiology reports on radiographic findings during follow-up (FU) after extremity fractures are generally not yet available when the patient is seen by the treating orthopedic trauma surgeon and may therefore be redundant. The aim of this study was to explore the inter-observer agreement on the reported findings of the FU radiograph between orthopedic trauma surgeons and radiologists.

**Method:**

This retrospective cohort study included all FU radiographs of adult patients with an extremity fracture, treated in a Dutch university hospital between January 2022 and July 2023. The radiologist's and orthopedic trauma surgeon's assessments of unacceptable alignment, delayed/non-union and abnormalities associated with osteosynthesis material (OSM) on the FU radiographs were collected from the medical files. Fracture healing was considered normal in the absence of these findings. The interobserver agreement of the radiographic parameters between surgeon and radiologist was determined using Cohens' Kappa (κ). Additionally, incidental findings and their clinical relevance were explored.

**Results:**

953 FU radiographs of 569 patients were included. The interobserver agreement was close to perfect for normal fracture healing (κ = 0.82, 95 % CI 0.77–0.88) and delayed/non-union (κ = 0.94, 95 % CI 0.89–0.99). The inter-observer agreement was substantial for unacceptable alignment of the fracture (κ = 0.80, 95 % CI 0.72–0.88) and abnormalities associated with the OSM (κ = 0.77, 95 % CI 0.65–0.89). Twenty-one incidental findings were diagnosed in the FU by the radiologist of which two were also independently described by the surgeon. Three of the findings that were missed or not described by the surgeon could have clinical significance, however the missing of these findings did not lead complications or additional hospital visits. These included two missed rib fractures and one osteochondral defect, which led to prolonged immobilization due to pain.

**Conclusion:**

Radiographic reports in the FU of extremity fractures have limited additional value for clinical care and probably lacks cost-effectiveness or efficiency.

## Highlights

• Radiologists and orthopedic trauma surgeons have an excellent agreement on the assessment of extremity fracture union.
• Incidental findings in fracture follow-up are rare; however, they are often not described or missed by orthopedic trauma surgeon.
• Incidental findings that are missed or not documented by the orthopedic trauma surgeon do not lead to complications.

## 1 Introduction

Radiographs are made during all phases of fracture care and are generally assessed by both the treating orthopedic trauma surgeon and the radiologist. After initial fracture treatment, radiographs are often made during follow-up (FU) visits to monitor the healing of the fracture. In clinical practice, the formal radiology report is not instantly made and therefore rarely available when the patient is seen by the treating orthopedic trauma surgeon. When the radiology report becomes available at a later moment, this will not be read by the treating surgeon. During these FU visits, the surgeon combines the clinical findings and his/her own radiographic assessment to decide on a treatment course. Due to all these unread radiology reports the question arose whether an orthopedic trauma surgeon is as competent as a radiologist in the assessment of fracture union of extremity fractures on radiographs and if clinically relevant complications and incidental findings are missed.

For specific fracture types and population groups the inter-observer agreement in the follow-up has been evaluated for radiologists and orthopedic (trauma) surgeons. The interobserver agreement on the general assessment of fracture healing in hip fractures between orthopedic trauma surgeons and radiologists was fair to moderate.^1^, ^2^, ^3^ Hannemann et al. also found moderate inter-observer agreement in radiographic assessment of scaphoid fracture healing.^4^ In patients with osteogenesis imperfecta an excellent inter-observer agreement was found for the assessment of the union of tibial fractures.^5^ Because the inter-observer agreement varies notably in these studies, it is essential to investigate whether these results are applicable to other fracture types as well.

The inter-observer agreement of radiographic assessment was investigated for multiple fracture types in healthy patients at the emergency department (ED). Discrepancies in assessment between the radiologists and clinical doctors of the ED were found in several studies, ranging from 2.5 to 11 % of the cases.^6^, ^7^, ^8^, ^9^, ^10^ In 44 % of these discrepancies, the treatment plan was changed after the secondary assessment by the radiologist.^6^ Hence, the assessment of fracture radiographs in the ED setting by the radiologist is essential for obtaining a correct diagnosis and for minimizing the risk of missing incidental findings. However, in the follow-up only the fracture union needs to be evaluated.

Incidental findings on FU radiographs obtained for evaluating fracture union are rare and can be missed. Velasco et al. found incidental findings in 2.1 % of radiographs ordered by orthopedic surgeons, for which monitoring was recommended in 42.2 %.^11^ However, we hypothesize that new clinically significant findings are unlikely to develop in the short interval between the fracture and FU radiograph.

Omitting the radiology reports of FU radiographs would lead to less workload for the radiology department. This is only justified, however, if the orthopedic trauma surgeon and the radiologist are equally competent in assessing the fracture union and complications and important additional findings are not missed, so that the quality of care is not compromised. Therefore, the aim of this study was to determine the inter-observer agreement on the reported findings of the radiographs made in the FU after initial treatment of extremity fractures between orthopedic trauma surgeons and radiologists. And, to determine the incidence and clinical relevance of additional or incidental findings on the FU radiographs that were missed by the orthopedic trauma surgeons.

## 2 Patients and methods

### 2.1 Study population

This retrospective cohort study included FU radiographs of all adult (≥18 y) patients with an extremity fracture who were treated in a university hospital in The Netherlands from January 2022 till July 2023. Clavicular fractures were considered to be extremity fractures. Fractures for which routine radiographic follow-up is not performed, such as those of the hand or foot, were excluded from this study. In accordance with local protocols of the university hospital, hip fractures are typically managed by orthopedic surgeons, whereas other fractures fall under the purview of the trauma surgery department. Consequently, hip fractures were also excluded.

FU radiographs were defined as radiographs made to assess the healing of the fracture, according to radiological standards performed in two perpendicular planes. Intraoperative radiographs and radiographs made within 72 h after the ED visit for further classification of the fracture were not included. Per patient, all radiographs taken at FU visits were analyzed. FU radiographs ordered by general practitioners were excluded. All fractures had a minimum follow-up of four months.

### 2.2 Data collection

All data was collected from the medical records, coded and stored in a Castor EDC database. The institutional Ethics Review Board approved the study and granted a waiver of obtaining informed consent.

### 2.3 Outcomes and other study parameters

For each FU radiograph the following parameters were scored based on the radiologist's and orthopedic trauma surgeon's written reports: unacceptable alignment, delayed or non-union and abnormalities associated with osteosynthesis material (OSM). Abnormalities with the OSM may include intra-articular placement, dislocation, or broken material. Overall fracture healing was defined as normal if no problems with the alignment, union or OSM were recorded. Additional traumatic findings to the baseline ED imaging were registered. Incidental findings were defined as findings not related to the fracture evaluated on the radiograph, with or without clinical consequence. Clinical consequence was defined as requiring additional imaging, further visits to the outpatient clinic or emergency department, or a (re)admission to the hospital. Trauma-related findings (e.g. luxation or swelling of the soft tissue), vascular calcifications, decreased bone mineralization or arthrosis were not registered as incidental findings.

Other study parameters were patient characteristics (sex, age), fracture site, primary treatment (cast immobilization or operation) and the cause of the fracture (trauma, insufficiency due to osteoporosis or pathological i.e. bone metastasis).

### 2.4 Statistical analysis

The data was analyzed using IBM SPSS Statistics for Windows, version 29 (IBM Corp., Armonk, N.Y., USA). Patient and fracture characteristics and outcome data were described using summary statistics. Differences in the radiographic parameters between surgeon and radiologist were tested using the McNemar test. The inter-observer agreement on radiographic parameters between surgeon and radiologist was assessed using Cohen's kappa (κ) with the corresponding 95 % confidence interval (CI). Kappa values ≤ 0 were interpreted as no agreement, 0.01–0.20 as slight, 0.21–0.40 as fair, 0.41–0.60 as moderate, 0.61–0.80 as substantial and 0.81–1.00 as almost perfect agreement.^12^

## 3 Results

656 eligible patients with a total of 1220 FU visits with imaging were identified. Computed tomography (CT; n = 38) and magnetic resonance imaging (MRI; n = 9) scans were excluded. 178 FU radiographs were not reported on in the medical files by both the surgery and radiology department and were excluded. Thirty-nine FU radiographs were also excluded because the surgeon had copied the text or conclusion from the radiology report. Another three FU radiographs were excluded because the surgeon had discussed these with the radiologist. In total, 953 FU radiographs of 569 patients were analyzed.

The study group included 205 males (36 %) and 364 females (64 %) with a mean age of 56.6 years (standard deviation 19.0). The most frequently depicted fracture site on the 953 radiographs was the radius and/or ulna (39.2 %; Table 1). 309 FU radiographs of fractures after operative treatment (32.4 %) and 644 of fractures treated with immobilization (67.6 %) were included. The number of FU radiographs per patient ranged between one and seven (mean 1.8). The length of FU ranged from 3 to 457 days (median 34).

**Table 1:** Characteristics of follow-up radiographs.

Radiographs in the follow-up N = 953	
Fracture site, n (%)	
Clavicle	68 (7.1)
Humerus	200 (21.0)
Radius/ulna	374 (39.2)
Femur	41 (4.3)
Tibia/fibula	270 (28.3)
Cause of fracture, n (%)	
Trauma	926 (97.2)
Insufficiency (osteoporosis)	7 (0.7)
Other pathology	20 (2.1)
Primary treatment, n (%)	
Cast immobilization	644 (67.6)
Operation	309 (32.4)
Number of radiographic visits per patient, median (IQR)	1 (1–2)
Period to follow-up, median days (IQR)	34 (9–79)

IQR – interquartile range.

The assessments of the radiographic parameters in the radiologist’ and surgeons’ reports are presented in Table 2. Inter-observer agreement was almost perfect for normal fracture healing (κ = 0.82, 95 % CI 0.77–0.88) and delayed-/non-union (κ = 0.94, 95 % CI 0.89–0.99). The agreement was substantial for unacceptable alignment (κ = 0.80, 95 % CI 0.72–0.88) and abnormalities associated with the OSM (κ = 0.77, 95 % CI 0.65–0.89). The McNemar test was not statistically significant for the assessment of all radiographic parameters. When performing the test separately for patients treated with cast immobilization and those who underwent surgery, the results remained statistically non-significant. Indicating that the radiologist and the surgeon were equally likely to report these deviations on the FU radiographs. When performing the McNemar test separately for groups receiving cast immobilization or patients an operation, the test remained not statistically significant.

**Table 2:** Agreement on radiographic parameters for fracture healing on follow-up radiographs between reports of the surgical and radiology departments.

	Surgeon N = 953	Radiologist N = 953	Cohen's Kappa	McNemar test
Normal fracture healing, n (%)	822 (86.3)	820 (86.0)	0.82 (0.77–0.88)	0.88
Unacceptable deviation, n (%)	62 (6.5)	58 (6.1)	0.80 (0.72–0.88)	0.52
Delayed-/non-union, n (%)	42 (4.4)	45 (4.7)	0.94 (0.89–0.99)	0.38
Abnormalities associated with the OSM, n (%)	29 (3.0)	33 (3.5)	0.77 (0.65–0.89)	0.42

OSM – osteosynthesis material.

In total 23 new incidental findings were identified in the radiology report (2.2 %), two of which were also reported by the orthopedic trauma surgeon (Table 3). Two of the 21 incidental findings not documented by the surgeon involved additional traumatic injuries beyond the evaluated fracture: one was a suspected avulsion fracture of the tertius, and one fracture of the ulnar styloid. Both patients recovered painlessly and regained the expected function due to the immobilization received for their larger fracture. The radiology department also identified two potential ligament ruptures – a lateral collateral ligament and a coracoacromial ligament – based on the observation of an enlarged gap between two bones near the fracture site. However, both patients had no clinical signs so that no additional diagnostics or interventions were performed. Fractured ribs were seen by the radiologist in one patient and suspected in another. Both patients did not receive additional treatment, and had an uncomplicated recovery. One osteochondral defect dorsolateral of the talus was detected by the radiologist and missed by the surgeon, however this patient received prolonged cast immobilization with an “ankle wrap” for analgesic reasons. One radiograph of a humerus showed an enlarged heart suggesting heart failure according to the radiologist; however, the patient had no symptoms associated with heart failure, therefore no additional diagnostics were performed. Furthermore, ten calcifications in soft tissue, two anatomical variants and one bone cyst were seen by the radiologist, however these had no clinical consequences.

**Table 3:** Number (%) of additional and incidental findings on 953 follow-up radiographs after extremity and clavicular fractures, according to the surgery and radiology departments.

Incidental findings	Surgeon N = 2		Radiologist N = 21	
Traumatic injury in addition to the ED radiograph	2 (0.2)∗		7 (0.7)∗∗	
Styloid fracture		1 (0.1)		1 (0.1)
Malleolus tertius fracture		1 (0.1)		1 (0.1)
Rib fracture (s)		–		2 (0.2)
Suspected rupture of ligament		–		2 (0.2)
Osteochondral defect		–		1 (0.1)
Bone cyst	–		1 (0.1)	
Calcification in soft tissues	–		10 (1.0)	
Anatomic variant	–		2 (0.2)	
Abnormality of an internal organ	–		1 (0.1)	

ED – Emergency Department. ∗and∗∗same/overlapping patients.

## 4 Discussion

The results of this study display an excellent inter-observer agreement between orthopedic trauma surgeons and radiologists for normal fracture healing in extremity fractures, as calculated by the Kappa value. In addition, radiologists do not report more cases of abnormal fracture healing, as determined by the McNemar test. Radiologists do report incidental findings more often. However, in our study, the incidental findings that were either missed or not documented by orthopedic trauma surgeons did not result in additional emergency department visits, hospital admissions, or any other complications noted in the patients' medical records. Following evaluation by a board-certified orthopedic trauma surgeon, these missed or unreported incidental findings were deemed to have no clinical significance. In our data no change in treatment plan occurred after the radiology report became available, and in retrospect the ‘missed incidental findings’ did not have significant influence on the patient's outcome. Based on our results, the radiology report in the FU of extremity fractures might be superfluous.

Other clinical trials that compared radiologists and surgeons in their assessment of fracture union found wide variation in inter-observer agreement ranging from 0.22 to 0.92 in multiple fracture sites and patient groups.^13^ Our results display an excellent inter-observer agreement, which might be explained in two ways. Firstly, the aim of our study was to determine whether the quality of care was compromised if there was no radiology report. Therefore, we investigated highly prevalent extremity fractures, while other studies focused on a single fracture site which was known to have radiological difficulties such as scaphoid fractures or operatively treated hip fractures. Or the inter-observer agreement was investigated in specific patient groups with bone diseases of complications, where the assessment of fracture union can be expected to be more complicated.^10^^,^^14^ Secondly, in most trials a scoring system was used where on multiple aspects of fracture union a score must be determined.^6^, ^7^, ^8^ While in our study written assessment (which sometimes consisted of only a few words) was deemed as normal fracture union in the absence of deviation, non-union or problems with the OSM.

The results of this study might have several implications for clinical practice. Omitting a report by a radiologist might lead to a reduction of total health care costs, considering the large incidence of fractures seen by orthopedic trauma surgeons. It could be argued that enhancing coordination between the surgical and radiology departments might reduce the number of “missed incidental findings.” However, our results indicate that the omission of these IF by the surgical department did not impact patient outcomes.

Additionally, the yearly increase in workload in radiology practices in highly specialized fields (CT and MRI) could benefit from this shift in work. Nevertheless, surgeons need to be aware that their responsibility increases, due to the fact that they have to interpret the complete radiograph for incidental findings. IF in the FU of extremity fractures are extremely rare, however, in case of doubt is direct accessible contact with a radiologist essential.

In 2012 there were over 176.000 extremity fractures in the Netherlands in patients aged >16 years with a rapidly growing absolute incidence.^15^ When assuming this increasing trend in incidence the number of extremity fractures in the Netherlands in patients over 16 years will be around 250.000 extremity fractures annually. In this study we found 1.8 FU moments with radiographs per patient, meaning roughly over 450.000 radiology reports in the Netherlands annually. Not reporting these radiographs will save the radiologists time, which could lead to a reduction of the total health care costs. Moreover, the time saved can be focused on specialized reporting of CT and MRI, showing an annual increase in utilization.^16^

Another aspect to consider are the medico-legal implications of omitting radiology reports in trauma care, particularly when potential litigation or damage claims are involved. In legal context, the reports serve as a critical piece of evidence, as they document the findings and conclusions of medical imaging. Without these reports, the legal standing of medical decisions may be compromised, especially when an adverse event arises from missed or overlooked findings. It could be argued that the emergency department (ED) radiograph report might suffice, or that follow-up radiographs could be retrospectively assessed by a radiologist if necessary. However retrospective review may be viewed as an after-the-fact attempt to rectify an omission or misinterpretation, which may not withstand legal scrutiny. Another critical medico-legal concern arises when non-trauma-related incidental findings are overlooked by the surgical department. In such scenarios, it seems reasonable that the surgical department could be held accountable, particularly if the hospital has chosen to omit radiology reports during the follow-up of extremity fractures. Whether this would result in different clinical outcomes can be argued, since in current practice the radiology report is often not read by the surgeon and in our data the ‘missed incidental findings’ had no clinical consequence. If radiology reports during the follow-up of trauma patients would be omitted and instead performed only when indicated by the orthopedic trauma surgeon, it is essential for the hospital to establish clear policies for the handling of IF and documentation of radiographs by orthopedic trauma surgeons.

It is essential to acknowledge the limitations of our study. Firstly, our study was single-center and conducted in a university hospital. The outcomes of research conducted in a single-center can be biased by the expertise of the medical staff, available resources, and local protocols of the hospital. University hospitals generally treat more complex and rare cases, meaning the cohort might not reflect the typical population. Both factors might limit the generalizability of our findings. Also, all radiographic assessments as written down in the radiology report and surgical notes are analyzed by a single observer, introducing potential bias. Additionally, 178 FU moments could not be included in the analysis because there was no written conclusion of the radiograph, and thirty-nine radiographs were excluded due to a copied radiology report by an orthopedic trauma surgeon, both of which introduced potential selection bias. The patients for whom an assessment of the radiograph is absent or copied could represent a subgroup of trauma patients. It can be presumed that patients with unacceptable alignment of the fracture are less likely to have no written assessment from the orthopedic trauma surgeon. It can also be argued that when there is no written or a copied conclusion the orthopedic trauma surgeon did not assess the radiograph itself. However, it is considered standard practice to evaluate the medical images when treating the patient. Additional discussions between the surgical and radiology departments without documentation in the medical record seem unlikely, as physicians typically consult within their own specialty when faced with uncertainty. In cases where interdisciplinary consultation is sought, it can be assumed that such discussions would be documented to provide evidence that the consultation took place. Moreover, our analysis also included FU radiographs assessed by residents and physician assistants under supervision of a board-certified orthopedic trauma surgeon or radiologist, not taking clinical experience into account. Lastly, the retrospective nature of our study may introduce bias and makes it impossible to determine whether the radiology report was available at the moment.

Our results seem to indicate that the radiology report might be superfluous; however, our study had several limitations. Therefore, a well-executed prospective multi-center trial, including both academic and non-academic hospitals, is needed to evaluate the inter-observer agreement between radiologists and orthopedic trauma surgeons, where both groups are instructed to assess fracture healing and possible incidental findings, minimizing the missing data. If such a study can duplicate our results, it would confirm and strengthen our conclusions.

## 5 Conclusions

Radiologists and orthopedic trauma surgeons have an excellent agreement in the assessment of FU radiographs of extremity fractures. Moreover, incidental findings as described in the FU phase did not lead to complications. Because the orthopedic trauma surgeon already evaluated the radiograph during the follow-up phase, the radiology report might be superfluous for the quality of care.

## CRediT authorship contribution statement

**Mare M.H. Walraven:** Conceptualization, Methodology, Formal analysis, Investigation, Writing – original draft. **Pieta Krijnen:** Conceptualization, Methodology, Writing – review & editing. **David A. Vuijk:** Writing – review & editing. **Monique Reijnierse:** Writing – review & editing. **Inger B. Schipper:** Writing – review & editing. **Marco F. Termaat:** Conceptualization, Methodology, Writing – review & editing, Supervision.

## Patient consent statement

Due to the retrospective nature of this study and the large number of patients included, individual patient consent was not obtained. The institutional Ethics Review Board approved the study and granted a waiver of obtaining informed consent. All procedures followed in this study were in accordance with ethical standards, including the principles outlined in the WMA Declaration of Helsinki.

## Ethical statement

The research described in this manuscript has been conducted in accordance with the ethical principles outlined in *The Code of Ethics of the World Medical Association* (Declaration of Helsinki). The manuscript also adheres to the *Recommendations for the Conduct, Reporting, Editing, and Publication of Scholarly Work in Medical Journals*.

## Funding statement

This research did not receive any specific grant from funding agencies in the public, commercial, or not-for-profit sectors.

## Declaration of competing interest

The authors declare that they have no known competing financial interests or personal relationships that could have appeared to influence the work reported in this paper.
